# Effect of various concentrations of superabsorbent polymers on soil particle-size distribution and evaporation with sand mulching

**DOI:** 10.1038/s41598-019-39412-x

**Published:** 2019-03-05

**Authors:** Wenju Zhao, Taohong Cao, Pinxin Dou, Jie Sheng, Minqiang Luo

**Affiliations:** 1 Coll. of Energy and Power Engineering, Lanzhou University of Technology, Lanzhou, 730050 China; 2 Northeast Agricultural University, Key Laboratory of Efficient Use of Agricultural Water Resources, Ministry of Agriculture, Haerbin Shi, P. R. China

## Abstract

Superabsorbent polymers (SAPs) are type of hydrogels capable to swell and absorb a large amount of water, but easily decomposed and oxidized by the air. We used electron-microscopic imaging in an indoor simulation with sand mulching to test the effects of various SAP concentrations on controlling evaporation and salt formation. The treatments were sand-mulched columns containing 0, 0.1, 0.2, 0.5 and 1.0% SAP. The soil particle pores were from dense to sparse and the corresponding fractal dimension decreased as SAP concentration increased. SAP concentration was correlated negatively with fractal dimension, clay-particle fraction and silt-volume fraction. And it showed a positive correlation with sand volume fraction. SAP concentration significantly affected the particle-size distribution. Water-storage capacity increased in each column layer (five 8-cm layers) at the same infiltration depth. Evaporation decreased the water content of each layer. Sand mulching combined with the SAP decreased evaporation in each layer relative to the control, which retained more water and decreased the accumulation of surface salt in the order 1.0% > 0.5% > 0.2% > 0.1% > 0. Salt migrated at 0–30 cm with sand mulching but 0–25 cm with sand mulching and SAP amendment. The decrease in salt accumulation was most effective at a SAP concentration of 0.2%.

## Introduction

Superabsorbent polymers (SAPs) are weakly cross-link functional polymeric materials with strong hydrophilic groups, which can absorb a large amount of water during a short time and the absorbed water is hardly removable even under pressure^1^. The application of SAP to soil is conducive to improving rainwater utilization efficiency in dry farmland^2^. And SAPs with high swelling capacity are of special interest as potential water retainer systems for agriculture fields^3^. SAP can absorb a solution of 10-folds to 1000-folds as much as its own weight^4^. SAP increased the water retaining capacity of soil sections where the suction pressure was between 0 and 3,000 cm^5^. The SAP can improve plant drought resistance and have been described as agricultural “micro-reservoirs”^6,7^. Goebel *et al*.^8^ reported that SAP can increase the water-holding capacity of soil and soil aggregation and aid the protection of organic matter. Woodhouse *et al*.^9^ found that SAPs could inhibit soil-water evaporation. Han *et al*.^10,11^ concluded that after SAP treatment, the soil hydraulic parameters and water holding capacity have undergone significant changes. The extensive use of SAPs, however, is severely restricted, because they are easily decomposed and oxidized by the air^12^.

Sand mulching can insulate soil from high illumination and hinder SAP oxidation to some extent, which have allowed the application of SAPs in northwestern China. Sand mulching can inhibit the evaporation of soil water, improve the soil environment, increase the infiltration of rainwater, store water and retain soil water^13–15^. So, studies have begun to investigate the effect of SAPs on sand-mulched soil. Zhao *et al*.^16^ studied the effect of various concentrations of SAPs on the infiltration of soil water with sand mulching, and found that the infiltration rate of soil water was optimal at a SAP concentration of 0.2%, which effectively increased the amount of water in the soil. However, there is little research on the effect of the combination of the two on soil evaporation, and the mechanism of action is unclear. Han and Yang *et al*.^17^ concluded that application of SAP can reduce soil bulk density, improve soil permeability, and cause soil expansion. Wang *et al*.^18^ found that the inhibition of soil-water evaporation did not improve if SAP concentrations were too high or too low. We therefore are trying to study the effect of SAPs on particle-size distribution and soil-water evaporation using indoor simulations and electron-microscopic imaging.

The purposes of this study were to determine (1) how combining a modern SAP with traditional sand mulching could inhibit soil-water evaporation and salt formation, (2) how different SAP concentrations with sand mulching affected soil particle-size distribution by using electron-microscopic imaging, (3) the best SAP concentration for inhibiting soil-water evaporation and salt formation is 0.2%.

## Materials and Methods

### Experimental Materials

The SAP used in the experiment was a superabsorbent polymer, a white granular dry powder with particle sizes of 0.8–1.0 mm. The main components were acrylamide copolymerized with convex/concave rod graft polymers. The soil, a sandy loam, was collected from a depth of 0–30 cm near the test site of Lanzhou University of Technology in Jingtai County, China. The soil was air-dried, crushed and sieved through a 2-mm sieve to remove impurities (the initial SWC was 0.75%). The particle-size distributions of the soil and the sand used for mulching are shown in Table 1.

**Table 1:** Particle-size distributions of the experimental soil and sand mulch.

soil Particle size (μm)	<1	<10	<50	<1000	<2000
soil (%)	10.06	23.13	80.26	99.46	100.00
Sand composition (mm)	<0.63	<1.25	<2.50	<5.00	<10.00
sand (%)	34.62	59.70	79.58	94.51	100.00

### Experimental design

**Figure 1:**
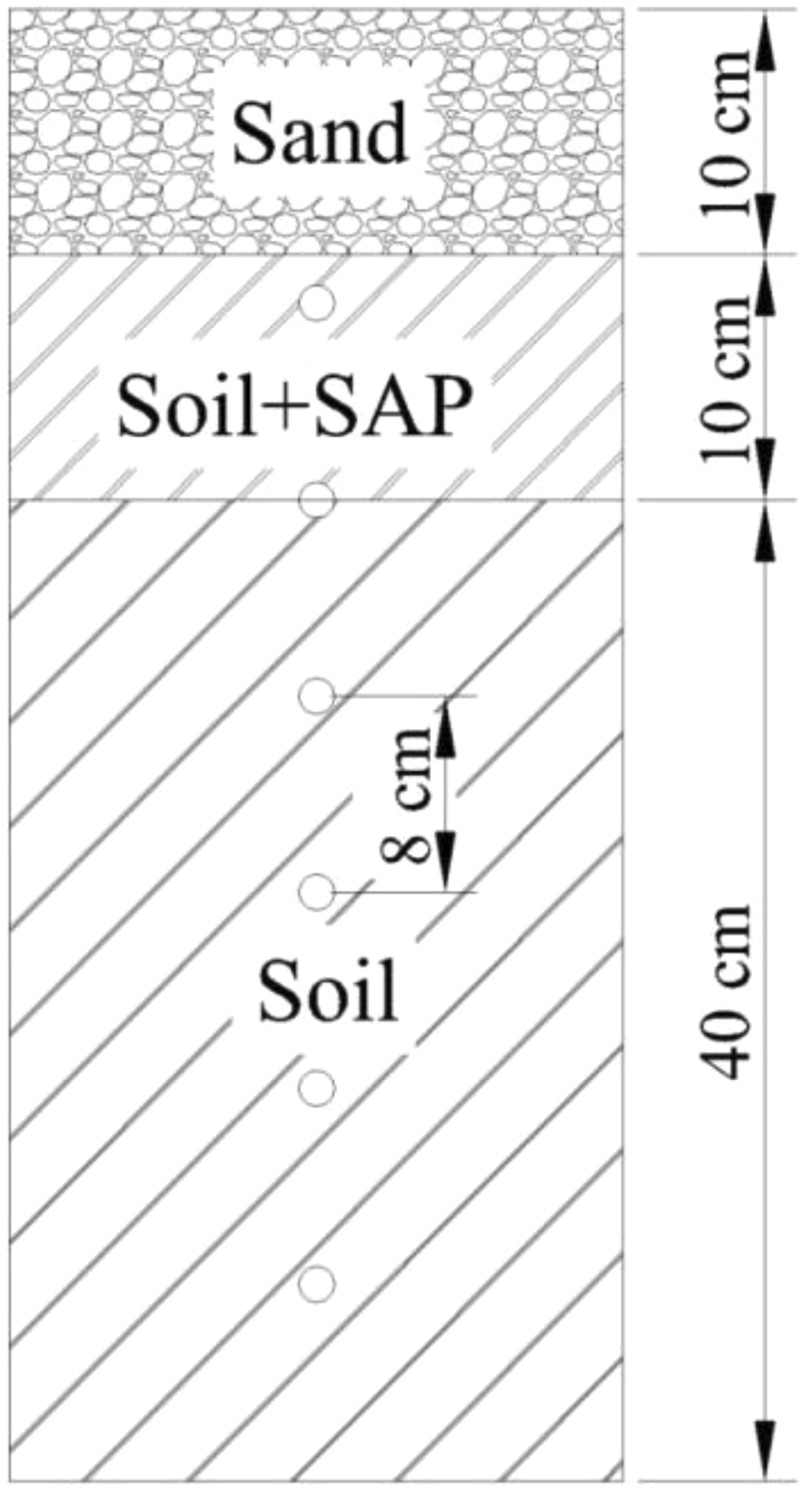
Soil column.

We tested a control group and four treatment groups. No SAP was applied to the soil in the control sample. SAP at a polymer concentration of 0.1, 0.2, 0.5, and 1% were respectively applied to the soil of treatment groups. The SAP concentrations were calculated as the mass ratio of the SAP to the mass of a 0–10 cm soil layer. A 10-cm layer of the sand mulch was then added, and the column was allowed to settle for 24 h. The soil columns were cylindrical Plexiglas columns, 70 cm high and 20 cm in diameter. The middle of the bottom of the column had a 2.5-cm circular hole (Fig. 1). The wall of the column contained six holes 2.5 cm in diameter and 8 cm apart vertically, which were plugged with wood when the soil was added. The bottom hole was covered with three layers of gauze to prevent the loss of soil. Soil (bulk density of 1.30 g/cm^3^) was added to the columns in 8-cm layers to a depth of 40 cm to maintain a consistent bulk density.

Using the Markov bottle for thin layer water experiment, after the infiltration, the soil standing for 48 h and then sampling, the sampling position were at the soil surface was 2, 10, 18, 26, 34, 42 cm, salt content and water content was measured as the initial data. After infiltration taken 0–10 cm soil surface, The Dried soil samples were scanned by soil particle scanning electron microscope S-48005.0 kV, the soil particle size distribution (PSD) was determined by the MS-2000 laser particle size analyzer. In the evaporation test, using a 275 W infrared lamp, the distance between the sand surface and the lamp was 20 cm, and evaporation was continuous 24 h/d for 30 d. The test environment temperature is controlled at 23 °C ± 2 °C. The relative humidity is 45% to 75%. Ten-gram samples were collected from the holes in the column walls the day before the beginning of evaporation and on days 10, 20 and 30 after the beginning of evaporation. The sampling position is the same as before the start of evaporation, and then filled the soil with the same water content. Soil water content (SWC) was determined by weighing the soil before and after drying. Soil salinity (1:5 soil: water) was measured by a conductivity meter (FG3-ELK, Mettler, Switzerland).

### Data processing

Particle sizes were graded using the USA grading standard: clay (0–2 μm), silt (2–50 μm) and sand (50–2000 μm). The particle sizes were set to 0–2, 2–5, 5–20, 20–50, 50–500 and 500–1000 μm for calculating the fractal dimension.

The fractal dimension of the particle-size distribution was calculated following Tyler *et al*.^19^:

1 $$\frac{V(r < {R}_{i})}{{V}_{T}}={(\frac{{R}_{i}}{{R}_{\max }})}^{3-D}$$

Both sides of Eq. (1) were log-transformed:

2 $$\mathrm{lg}[\frac{V(r < {R}_{i})}{{V}_{T}}]=(3-D)\mathrm{lg}(\frac{{R}_{i}}{{R}_{{\rm{\max }}}})$$

Where *r* is particle radius, *R*_*i*_ is particle size of grade *i*, *V(r* < *R*_*i*_) is particle size less than *R*_*i*_, *V*_*T*_ is total particle volume and *R*_*max*_ is the largest particle size. The left side of Eq. (2) was used as the y-axis of a scatter plot, and the right side was used as the x-axis, with the least squares method used for linear fitting.

All data were analyzed using Excel (version 2010, Microsoft Corporation, Redmond, USA), IBM SPSS Statistics (version 20.0, International Business Machines Corporation, Armonk, USA) and Origin (version 8.0, OriginLab, Hampton, USA).

## Results and Discussion

### Effects of various SAP concentrations with sand mulching on soil particle-size distribution

**Figure 2:**
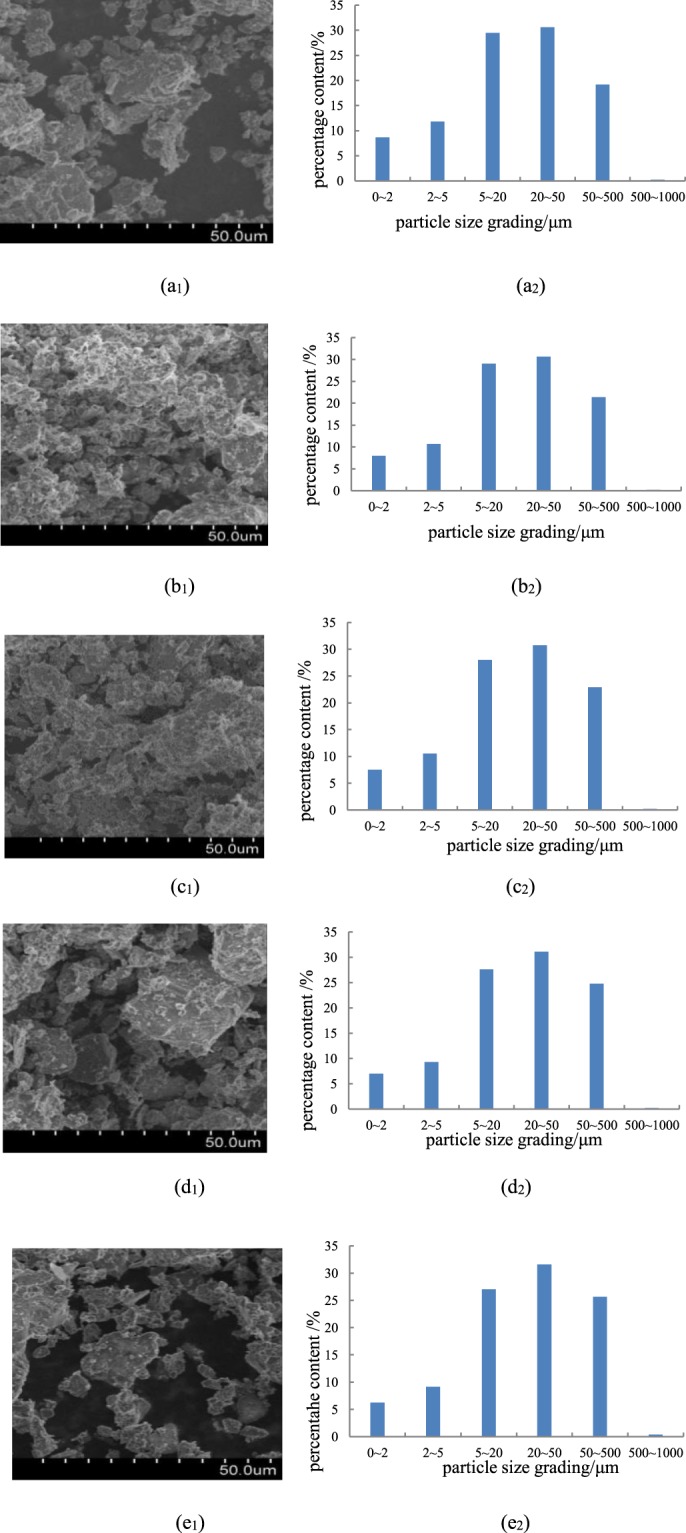
Image and distribution of soil particle size. (**a**_**1**_,**a**_**2**_**)** particle size distribution of CK; (**b**_**1**_,**b**_**2**_**)** particle size distribution with SAP concentration of 0.1%; (**c**_**1**_,**c**_**2**_) particle size distribution with SAP concentration of 0.2%; (**d**_**1**_,**d**_**2**_) particle size distribution with SAP concentration of 0.5%; (**e**_**1**_,**e**_**2**_) particle size distribution with SAP concentration of 1.0%.

Because of the strong water absorption properties of SAP, sandy soil treated with SAP the soil particle size distribution has changed and thus the soil structure has also changed^20^. Therefore, SAP was an important factor affecting soil particle-size distribution (PSD). The percentages of clay (0–2 μm), silt (2–50 μm) and sand (50–2000 μm) differed among the five test soils (Fig. 2) in the order silt > sand > clay particles. Clay is the largest percentage of particles, which was about 80%, sand content from 20 to 25%. From the particle size figure, the distribution of particles showed different density in different concentration of SAP. In the figure b_1_, c_1_, d_1_, the particles have smaller pore, in figure a_1_, e_1_, the soil particles have larger pore. Which showed is that increased with the concentration of SAP, the soil pore from dense to sparsely. The relationship between SAP concentration and the PSD of the surface soil with sand mulching is presented in Tables 2 and 3.

**Table 2:** Relationship between different SAP concentration and soil particle size distribution with sand mulching.

various SAP concentrations/%	Content of different particle sizes /%						SWC/%	fractal dimension D	the correlation coefficient/R^2^
	clay	sily			sand				
	<2 μm	2~5 μm	5~20 μm	20~50 μm	50~500 μm	>500 μm			
0	8.665	11.816	29.498	30.606	19.208	0.207	28.55	2.568	0.887
0.10	7.979	10.716	29.063	30.645	21.382	0.215	32.70	2.550	0.894
0.20	7.508	10.559	28.013	30.756	22.940	0.224	33.64	2.539	0.898
0.50	6.971	9.280	27.641	31.083	24.786	0.239	34.56	2.520	0.904
1.00	6.256	9.115	27.023	31.578	25.653	0.375	38.16	2.502	0.904

**Table 3:** Correlation between soil particle size and fractal dimension.

soil properties	D	the volume fraction of clay (%)	the volume fraction of sily (%)	the volume fraction of sand (%)	SAP concentrations (%)	SWC (%)
D	1					
the volume fraction of clay (%)	0.998^**^	1				
the volume fraction of sily (%)	0.973^**^	0.973^**^	1			
the volume fraction of sand (%)	−0.988^**^	−0.989^**^	−0.997^**^	1		
SAP concentrations (%)	−0.95^*^	−0.949^**^	−0.865	0.899^*^	1	
SWC (%)	−0.968^**^	−0.977^**^	−0.937^**^	−0.957^*^	0.911^*^	1

The fractal dimension (D) of particle size varied with the volume fractions of the particle sizes (Table 2). D gradually decreased as SAP concentration increased; fine-particle content decreased at high SAP concentrations. D was correlated positively with the volume fractions of clay and silt particles and negatively with the volume fraction of sand (Table 3). The volume fractions of silt and clay particles increased, and D tended to increase significantly, indicating that the non-uniformity of particle-size distribution increased with clay content. SAP concentration was correlated negatively with D and the volume fractions of clay and silt and positively with the sand volume fraction, indicating that the concentration of SAP could affect particle composition. SAP concentration was positively correlated with soil-water content, consistent with the water-storage characteristics of SAPs.

**Figure 3:**
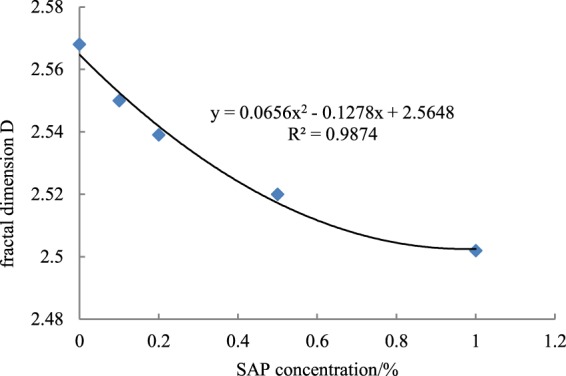
Correspondence between soil fractal dimension and SAP concentration.

The fractal dimension of particle size and the concentration of SAP were fitted with a quadratic function (Fig. 3). D decreased as SAP concentration increased, indicating that the clay content in the surface soil gradually decreased. The relationship between D and SAP concentration was consistent with a quadratic function:

3 $${\rm{y}}={{\rm{ax}}}^{2}+{\rm{bx}}+{\rm{c}}$$

Where a, b and c are constants. The coefficient of determination, *R*^2^, was 0.987.

The various concentrations of SAP had a significant effect on the particle-size distribution after infiltration. The particle pore was from dense to sparsely, D decreased as SAP concentration increased and clay content decreased at high SAP concentrations.

### Effects of various SAP concentrations with sand mulching on SWC

Due to the influence of soil water storage capacity on different concentration of SAP, SWC was difference under each layer. The sampling points at 2 cm (with SAP) and 18 cm (no SAP) as the representative point to research SWC on different concentrations of SAP, as shown in Table 4. The soil moisture content at 2 cm and 18 cm decreases with the evaporation process, which accords with the soil dehydration process. On different soil columns, the SWC with SAP is higher than CK, either before or after the evaporation, SWC increased with increasing concentrations of SAP, it shows that the water retention effect of SAP is 1.0% > 0.5% > 0.2% > 0.1% > CK.

**Table 4:** The average water content at different sampling in different SAP concentrations.

sampling points		CK	0.10%	0.20%	0.50%	1.00%
2 cm	before evaporation	36.73	37.83	39.29	40.66	42.55
	day 10 of evaporation	28.55	32.70	33.64	34.56	38.16
	day 20 of evaporation	27.97	29.41	31.57	32.08	34.64
	day 30 of evaporation	26.07	28.91	30.85	30.93	33.65
18 cm	before evaporation	34.33	36.07	37.52	37.84	38.81
	day 10 of evaporation	30.84	30.93	32.67	33.83	34.85
	day 20 of evaporation	29.21	29.67	31.25	31.84	33.13
	day 30 of evaporation	28.21	29.35	30.34	31.53	33.76

**Figure 4:**
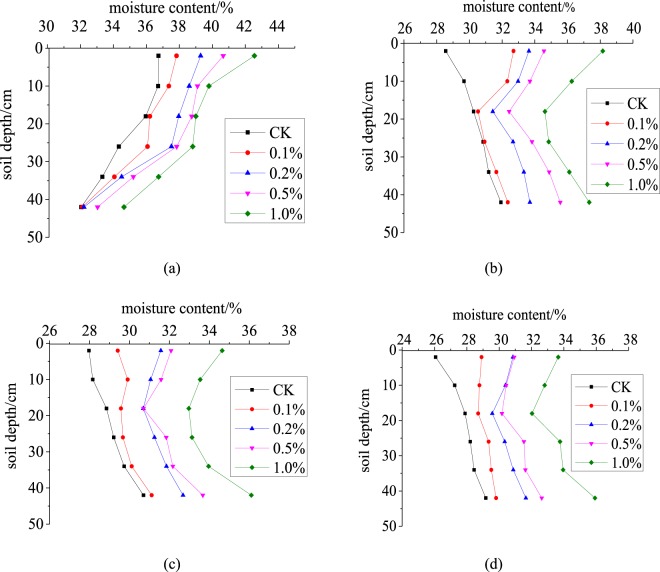
Soil-water content with depth for the various SAP concentrations. (**a**) before the beginning of evaporation; **(b)** day 10 of evaporation; **(c)** day 20 of evaporation; **(d)** day 30 of evaporation.

Soil water will be redistributed after infiltrating and standing for 48 h, and SWC will vary among the layers depending on the SAP concentration and the amount of evaporation. The SWC in each column reached saturation after 48 h of redistribution by gravitational force and capillary action and decreased with depth (Fig. 4a). The water-storage capacity differed among the columns due to the different SAP concentrations. SWC increased with SAP concentration. The average SWCs in the columns before evaporation were 34.85, 35.61, 36.73, 37.38 and 38.59% for the SAP concentrations of 0, 0.1%, 0.2%, 0.5% and 1.0%, respectively. The average SWCs in the columns with SAP concentrations of 0.1%, 0.2%, 0.5% and 1.0% were higher than the CK SWC by 0.76%, 1.88%, 2.53% and 3.74%, respectively. Both the sand and the soil mixed with the SAP could thus increase soil-water storage, which would reduce irrigation and increase the use of rainwater.

SWC of each layer decreased with the duration of evaporation (Fig. 4b–d). SWC of each layer decreased less in the columns containing the SAP than in CK after day 10 of evaporation. After 10 days of evaporation, SWC from top to bottom first decreased and then increased and was substantially lower in the middle than the upper layer, due to evaporation. The SAP will swell when it absorbs water and will form agglomerates, further cutting off the channels of rising water, because the SAP can absorb more water than the soil. The water lost from the upper layer by evaporation will be quickly supplemented by water from the lower layer, so water is lost faster from the middle than the upper layer. The lower layer is less affected by evaporation, so the moisture content of the lower layer decreased slowly. By day 30 of evaporation, when the concentration of SAP is 0.1%, 0.2% and 0.5%, the water content of upper and lower layers tends to be the same. The water content of each layer was highest at a SAP content of 1.0%, because less water is absorbed by the lower layer at SAP contents <0.5%. Most water is lost from the SAP layer. Soil with high contents of SAP (e.g. 1.0%) can absorb more water than the lower layer.

SWC was about 30% in each layer at a SAP concentration of 0.2%, which would be able to meet the needs of crops. SWC would be too high at SAP concentrations >0.2%, leading to lower rates of seed germination and poor economic returns.

### Effects of various SAP concentrations with sand mulching on salt content in soil profile

**Figure 5:**
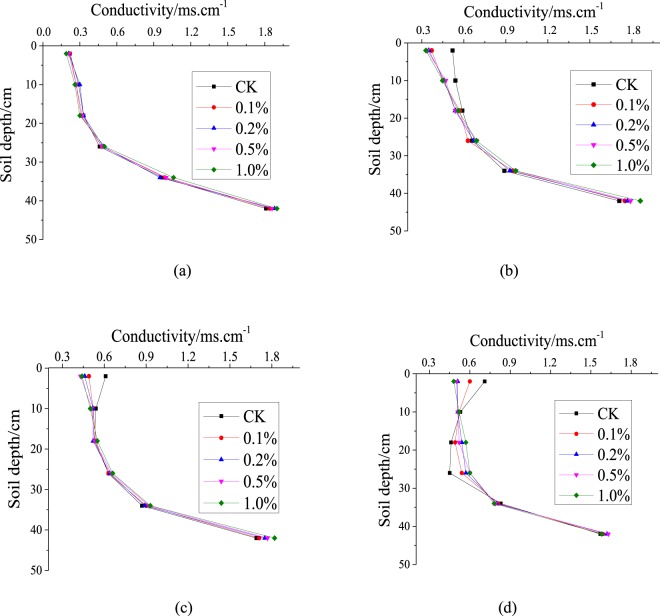
Variation of soil-salt content with depth for the various concentrations of the SAP. (**a**) before the beginning of evaporation; **(b)** day 10 of evaporation; **(c)** day 20 of evaporation; **(d)** day 30 of evaporation.

Salts are mainly transported by migrating water, so soil water is the main carrier of salt. Two processes are important in the accumulation of surface salt: the downward leaching of salt leading to the desalination of the surface soil, and the upward migration of salt in the water evaporated from the surface soil. The effects of different concentrations of SAP on soil average salt content were studied at 2 cm (with SAP) and 18 cm (no SAP) of sampling points (Table 5). Soil salinity varied over time in the columns due to evaporation (Fig. 5).

**Table 5:** The average salt content at different sampling in different SAP concentrations ms/cm.

sampling points		CK	0.10%	0.20%	0.50%	1%
2 cm	before evaporation	0.22	0.22	0.21	0.21	0.19
	day 10 of evaporation	0.52	0.37	0.35	0.34	0.33
	day 20 of evaporation	0.61	0.49	0.46	0.44	0.43
	day 30 of evaporation	0.71	0.60	0.51	0.48	0.50
18 cm	before evaporation	0.33	0.31	0.33	0.30	0.32
	day 10 of evaporation	0.59	0.57	0.54	0.54	0.56
	day 20 of evaporation	0.53	0.54	0.52	0.55	0.53
	day 30 of evaporation	0.46	0.49	0.54	0.57	0.52

It can be seen from Table 5 that the soil average salt content with the SAP is much lower than CK, and the change trend of the average salt content at 2 cm is generally increased with the progress of evaporation, but the trend at 18 cm is first increased and then decreased. This is consistent with soil salt accumulation in the surface. Whether it is evaporated before or after evaporation, the average salt content of the soil decreased with the increase of the concentration of the SAP. The results showed that the SAP could decrease the salt content, and the salt suppression effect was 1.0% > 0.5% > 0.2% > 0.1% > CK.

We simulated the two processes of salt transport in the soil columns (Fig. 5a). The salt contents before evaporation were 0.22, 0.22, 0.21, 0.21 and 0.19 ms/cm in the columns with SAP concentrations of 0, 0.1, 0.2, 0.5 and 1.0%, respectively. The average soil salt content was lower in the columns containing the SAP than in CK and decreased as SAP concentrations increased. The SAP in the upper layer formed an area that stored water, so that the salt in the soil was more fully dissolved in the water, and more salt was leached to the lower layers. Mixing soil with SAPs can thus leach salt downward. Salt was continuously concentrated in the surface layer during evaporation as water moved upward through the soil capillaries (Fig. 5b,c). The range of salt migration was only 0–30 cm with sand mulching but was 0–25 cm with the combination of sand mulching and SAP amendment. The salt contents in CK and 0.1% SAP were significantly lower in the middle layer than the upper and lower layers (Fig. 5d). More salt accumulated in CK than the 0.1% SAP treatment, due to the inhibition of evaporation by the SAP. Small amounts of salt accumulated in the 0.2, 0.5 and 1.0% SAP treatments. Adding a SAP to soil can thus effectively inhibit the accumulation of salt on the soil surface. The prevention of accumulation was optimal at a SAP concentration of 0.2%.

## Conclusion

1. The different SAP concentrations had a significant effect on the distribution of soil particle sizes. The fractal dimension decreased as the SAP concentration increased, and the particle pore was from dense to sparsely. SAP concentration was correlated negatively with fractal dimension, clay-particle fraction and silt volume fraction and positively with sand volume fraction. High SAP concentration decreased the clay content.
2. SAP mixed into the top 10 cm of soil increased the water-storage capacity of each layer of the sand-mulched soil column, which increased with concentration. Soil mixed with SAP significantly decreased the evaporation of soil water. A SAP concentration of 0.2% could decrease the evaporation of soil water enough to meet the needs for normal plant growth.
3. SAP mixed with soil increased desalination by increasing leaching in the order 1.0% > 0.5% > 0.2% > 0.1% > 0. SAP concentrations >0.2% did not significantly decrease salt formation further. Salt migrated 0–30 cm with sand mulching but 0–25 cm with sand mulching and SAP amendment. Mixing 0.2% SAP into the 0–10 cm soil layer decreased both the evaporation of soil water and salt formation. SAP amendment at this concentration thus represents an economically feasible strategy for increasing crop yield in arid areas.
